# Enormous Aneurysm of the Deep Femoral Artery: A Case Report

**DOI:** 10.7759/cureus.104795

**Published:** 2026-03-06

**Authors:** Nektarios Galanis, Christos Lyrtzis, Lamprini Navrozidou, Georgios Trikoilis, Pirro Majko, George Paraskevas

**Affiliations:** 1 Department of Anatomy, National and Kapodistrian University of Athens School of Medicine, Athens, GRC; 2 Department of Anatomy and Surgical Anatomy, Aristotle University of Thessaloniki, Thessaloniki, GRC

**Keywords:** aneurysm, case report, ct angiography, deep femoral artery, multiple myeloma

## Abstract

Isolated true aneurysms of the deep femoral artery (DFAA) are exceptionally rare clinical entities. This report aims to describe the diagnostic challenges and therapeutic dilemmas associated with a huge 5.3 cm isolated DFAA in a patient with coexisting systemic hematological malignancy. A 77-year-old male patient with multiple myeloma underwent radiographic investigation for skeletal pain, which incidentally identified a 5.3 cm isolated left DFAA. Multi-planar CT angiography (CTA) characterized the lesion as a 10-cm long fusiform aneurysm featuring significant mural thrombus (3.7 cm thick) and irregular luminal ulcerations. Given the high risk of rupture, open surgical excision and ligation were recommended. However, following comprehensive clinical counseling regarding the natural history of the disease, the patient opted for conservative management with watchful waiting. Isolated DFAAs of this magnitude are rare and may be clinically masked by systemic pathologies such as multiple myeloma. This case highlights the diagnostic utility of CTA in characterizing complex vascular lesions and illustrates the ethical challenges that arise when patients decline indicated surgical treatment.

## Introduction

The deep femoral artery (DFA), also known as the profunda femoris artery, is the largest branch of the femoral artery and its main function is to supply blood to the skin of the medial thigh region, the proximal femur and the muscles that extend, flex and adduct the thigh. It is located deep within the thigh and originates below the inguinal ligament from the posterolateral aspect of the femoral artery. It travels then between the pectineus and adductor longus muscles, and passes between the adductor longus and adductor brevis muscles, pierces the adductor magnus and ends by anastomosing with the muscular branches of the popliteal artery. Along its course, the DFA gives off several branches: the lateral circumflex femoral artery, which is the first branch and courses laterally, wrapping around the proximal femur; the medial circumflex femoral artery, which passes around the posterior aspect of the femur; and the perforating femoral arteries, which pierce the thigh [1]. At its origin, the DFA has a diameter of approximately 5 to 6 mm (range 4 to 9 mm) [2]. Femoral artery aneurysms are generally classified by anatomy as true or false (pseudoaneurysms). True aneurysms involve a localized dilation of all three vessel wall layers, intima, media, and adventitia, surpassing the normal vessel diameter by more than 50% [3]. While the common femoral artery is the most frequent site of involvement (57%), isolated true aneurysms of the DFA (expansion of the original diameter more than 1.5 times) are exceptionally rare clinical entities. According to updated literature, they account for only 0.5% of all peripheral aneurysms and between 1% and 2.6% of all femoral aneurysms [4-6]. Due to their deep anatomical location beneath the adductor muscles, these aneurysms often remain clinically silent, leading to a high incidence of rupture as the primary clinical presentation, ranging from 18% to 45% in various series [4,7]. This underscores the life-threatening nature of this pathology despite its rarity. Small aneurysms often remain undetected for a long time. The disease is usually asymptomatic and causes discomfort only when the aneurysm becomes very large. These symptoms can include local or generalized leg swelling, numbness, pain, or toe ischemia [4]. We present the case of a mainly asymptomatic huge aneurysm of the left DFA. Given this extreme rarity, documenting such cases is vital for establishing standardized diagnostic and management protocols. This case report has been reported in line with the Surgical CAse REport (SCARE) checklist [8].

## Case presentation

**Figure 1:**
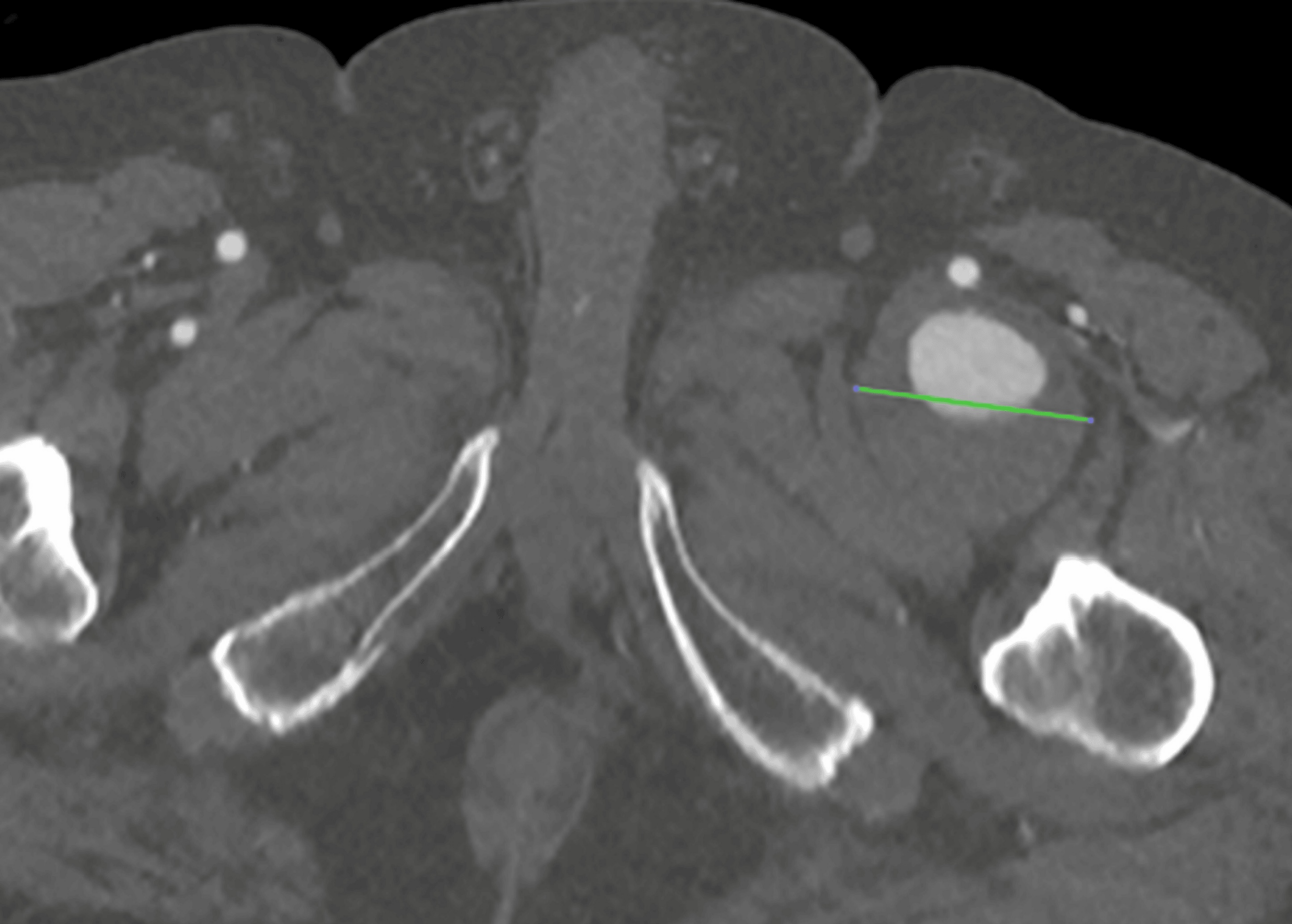
CT angiography, transverse plane It shows the left deep femoral artery aneurysm in the proximal third part of the vessel (transverse size: 5.1 cm - green line) with mural thrombus.

**Figure 2:**
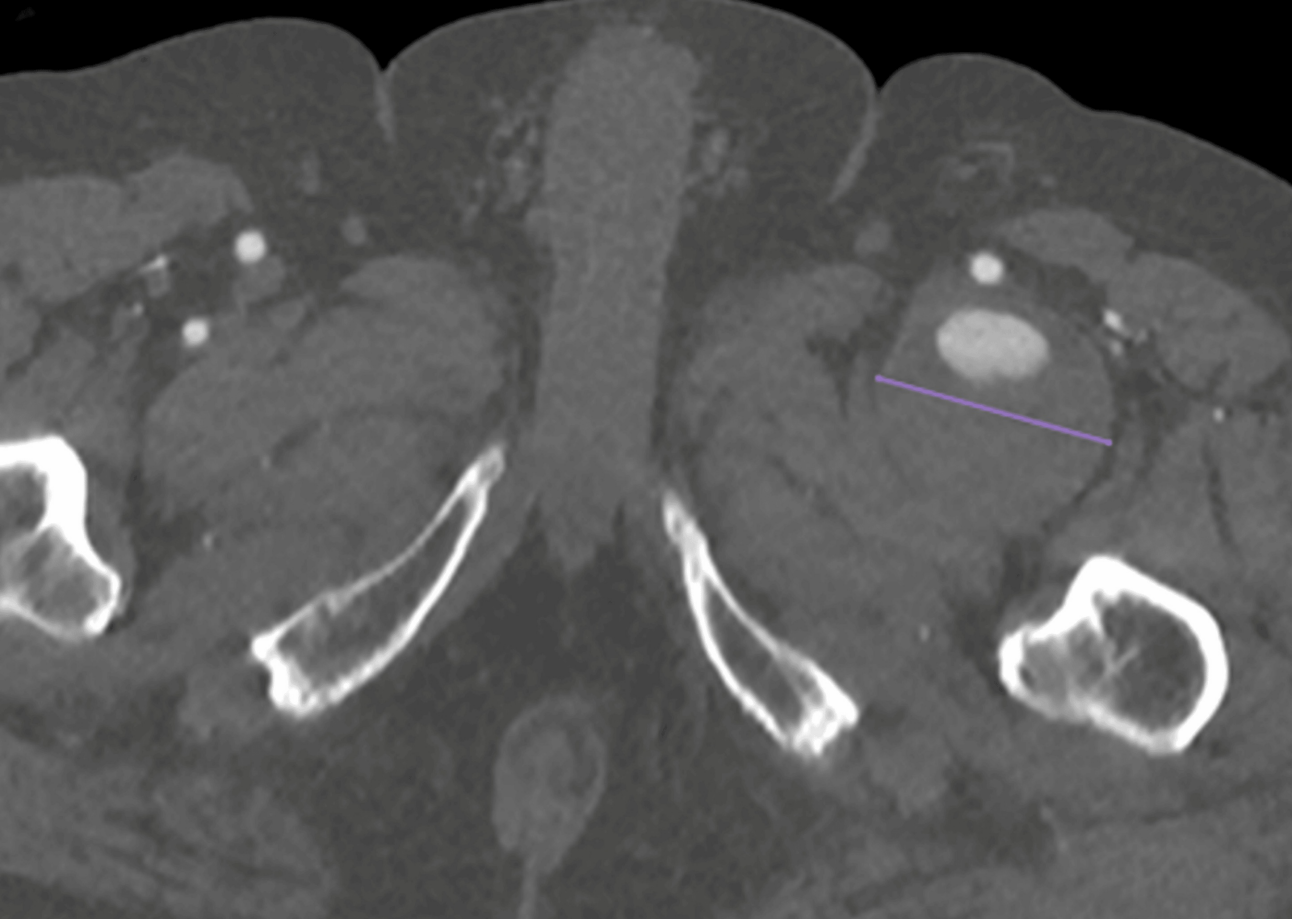
CT angiography, transverse plane It shows the left deep femoral artery aneurysm in the middle of the pathology (transverse size: 5.3 cm - purple line) with a thick mural thrombus.

**Figure 3:**
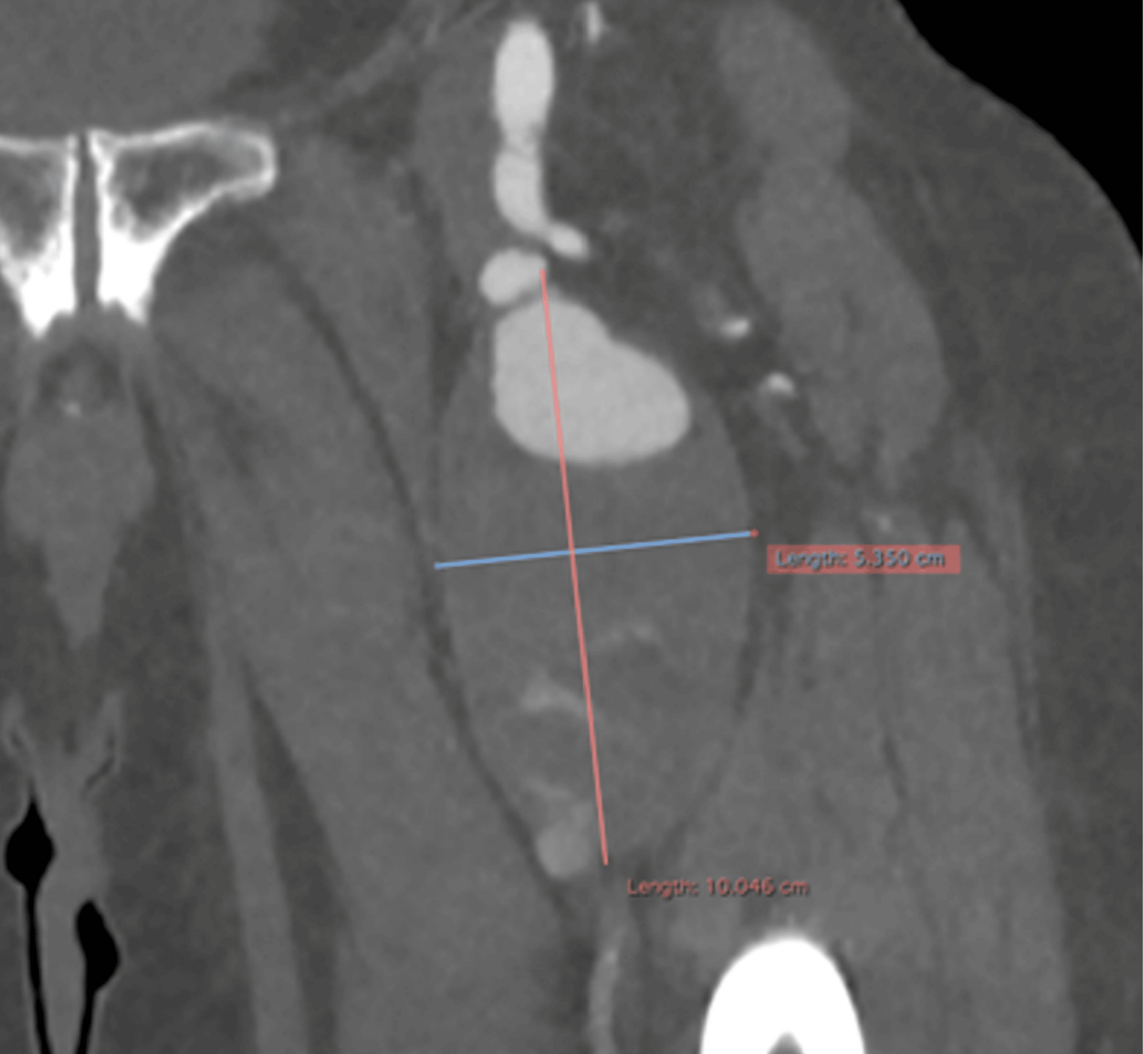
CT angiography, frontal plane It shows the left deep femoral artery aneurysm with its craniocaudal and transverse extension and irregular mural thrombus.

**Figure 4:**
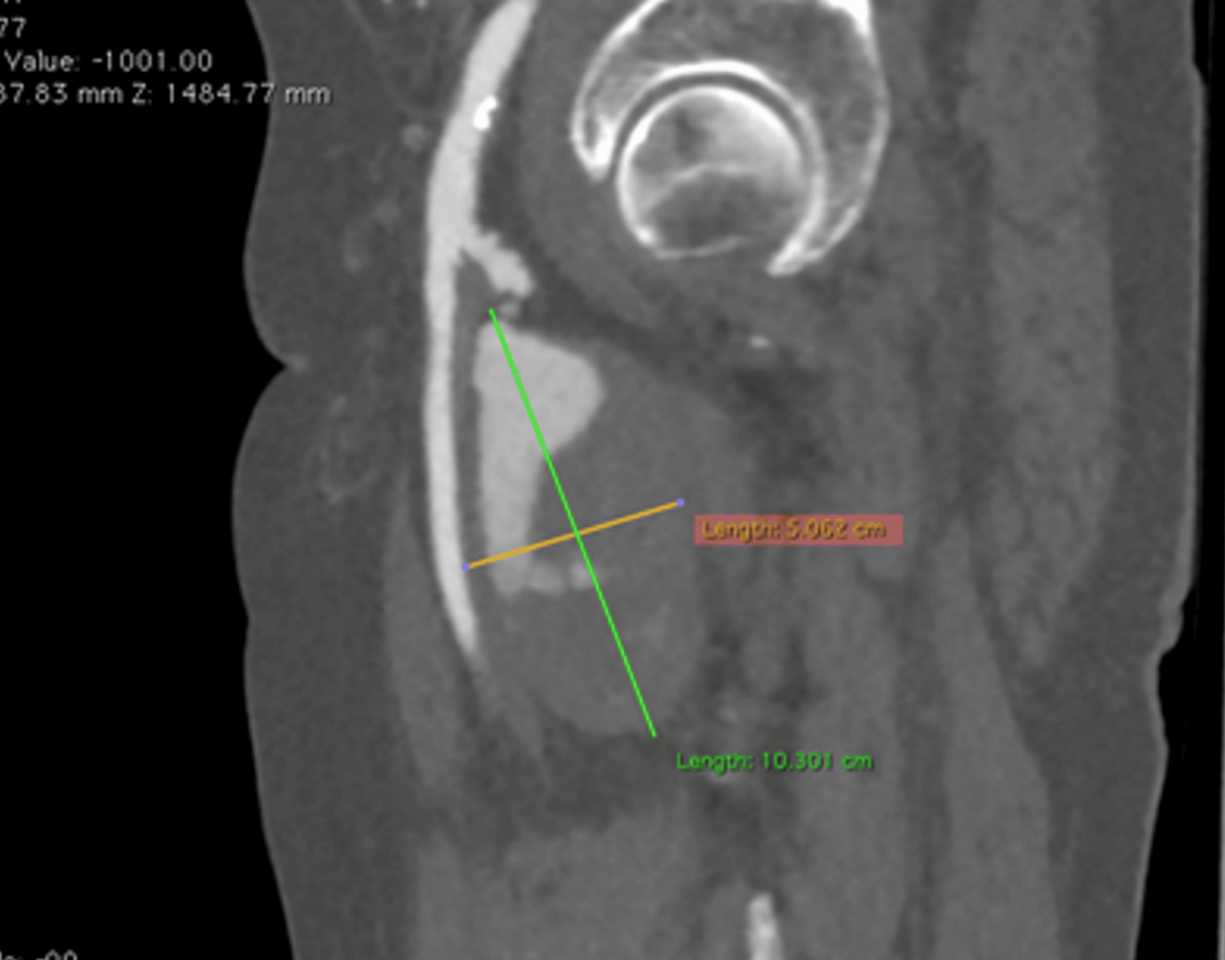
CT angiography, sagittal plane It shows the left deep femoral artery aneurysm with its craniocaudal and anteroposterior extension. Note the irregular mural thrombus, possibly due to multiple ulcerations.

A 77-year-old male patient was diagnosed with multiple myeloma in 2023. At that time, a general weakness had led to the investigation and confirmation of the diagnosis. Although he underwent immunotherapy, the patient maintained good condition and was self-sufficient. He also had cardiovascular risk factors, such as hypertension and hyperlipidemia, which were under sufficient medical control. At the end of 2024, he experienced increasing bone and joint pain, especially in the pelvic and proximal femoral area. Consequently, an ultrasound examination was performed, which surprisingly revealed a ca. 5 cm diameter aneurysm of the left DFA, since there were no relative clinical signs such as bright pulses or palpable pulsatile mass prior to this examination. The aneurysm was classified as asymptomatic because similar symptoms were also felt on the right side with the same onset and characteristics. A CT angiography from the infrarenal aorta to the foot arteries was performed promptly. The examination revealed a large fusiform aneurysm in the left proximal DFA with a craniocaudal length of around 10 cm, an anteroposterior diameter of around 5.1 cm and a transverse diameter of around 5.3 cm with the presence of mural thrombus of maximum posterior thickness around 3.7 cm. The edge of the lumen appeared irregular, probably in the context of multiple ulcerations (Figures 1-4).

Despite the aneurysm being classified as asymptomatic, we recommended surgical treatment to the patient after thorough consultation, because we estimated the possibility of rupture to be very high due to its size. We did not consider endovascular exclusion of the aneurysm to be a sensible option with a high degree of technical success. Our surgical treatment recommendation consisted of a completely open excision of the aneurysm with ligation of the side branches, without a vascular reconstruction due to the very good collateralization and the excessively long operating time a reconstruction would need. After the patient was informed about the benefits, but also the risks and dangers of the planned surgery, he decided against it. Despite intensive communication efforts, we were unsuccessful in integrating him into a follow-up plan. To our knowledge, the patient is still untreated.

The patient completely denied further follow-up examinations since he had decided against surgical therapy. This left us without information about his current status. In the last recruitment telephone communication, the patient described his general condition as “stable”, although he remains untreated to our knowledge. During the counseling sessions, when information about the disease and therapeutic options were given, the patient - accompanied by his wife - was fully aware of the dangers and the potentially fatal risk of rupture. However, he expressed a greater fear of possible perioperative adverse events. Thus, respecting the principle of patient autonomy, we did not feel ethically authorized to put further pressure on him and his environment. This underscores the ethical challenge of managing high-risk vascular pathologies when a mentally competent patient exercises their right to refuse indicated treatment.

## Discussion

Profunda femoris artery aneurysms are rare, accounting for roughly 140 case reports in the English literature, as of 2012 [4]. While the majority of femoral aneurysms involve the common femoral artery, our case represents a significant departure from this typical anatomical distribution. Isolated involvement of the DFA is documented in only a small fraction (1% to 2.6%) of all femoral cases, making it a distinct clinical outlier compared to more frequent sites [5,6]. Our case is particularly notable not only for the sheer size of the aneurysm (5.3 cm) but also for its presentation as an isolated pathology. This contrasts with the typical presentation, where femoral aneurysms are often a manifestation of systemic arteriopathy. Approximately 50-90% of patients present with concomitant abdominal aortic aneurysms [9].

The etiology of DFAA is predominantly atherosclerotic (reported in 82% of cases), though etiologies may vary [10]. Other causes such as trauma, infection, and disorders accompanied by inflammation, such as Behçet’s disease, acromegaly, or Marfan Syndrome place patients at higher risk [3]. Uniquely, our patient presented with a history of multiple myeloma. Although not considered an inherently occlusive disorder, it is the second most common hematological malignancy, biomarkers of which include hypercalcemia, renal failure, anemia, and bone lesions due to infiltration of plasma cells into the marrow [11]. This complex co-morbidity makes our case a unique clinical challenge, as the skeletal pain masked possible vascular symptoms.

Clinical Presentation

Because of the DFA's deep location beneath the adductor muscles, aneurysms can grow extensively while remaining undetected. This expansion can lead to dangerous mass effects; specifically, large aneurysms can compress the adjacent femoral vein, causing deep vein thrombosis (DVT) and subsequent pulmonary embolism (PE), which are life threatening complications [12]. Indeed, up to 40% of patients are asymptomatic at the time of diagnosis, and when symptoms do occur, they are often non-specific [13]. The differential diagnosis for a groin mass or pain in this region is broad, including inguinal hernia, lymphadenopathy, saphena varix, and soft tissue neoplasms [14]. Similarly, our patient experienced increasing bone and joint pain, especially in the pelvic and proximal femoral area. However, these symptoms were not classified as characteristic to this case due to similar pain on the side contralateral to the aneurysm. This clinical 'masking' was further compounded by the patient's underlying multiple myeloma, which typically presents bilateral skeletal involvement. Moreover, the deep anatomical location of the DFA meant that even a 5.3 cm aneurysm did not present with a clearly pulsatile mass, and the preservation of distal pulses (popliteal and pedal) initially diverted suspicion away from an acute vascular event.

Computed tomography angiography (CTA) remains the gold standard for a definitive diagnosis [15]. It provides precise vessel sizing and, more importantly, characterizes the intraluminal thrombus, as ultrasound and MRI are often unable to distinguish between a partially thrombosed aneurysm and a soft tissue tumor [16]. We previously established that true aneurysms 1.5 times larger in diameter than the ordinary range of 4 to 9 mm are uncommon. Sizes may vary even among these rarer, larger cases. In a study by the Mayo Clinic detailing 15 patients with 17 profunda femoris aneurysms, the reported mean diameter was 3.4 cm, with a range of 1.5-7.5 cm [10]. Our case falls above their reported mean. Our CTA findings show our patient’s aneurysm growing to a massive craniocaudal length of 10 cm, an anteroposterior diameter of around 5.1 cm, and a transverse diameter of around 5.3 cm. Moreover, in our patient, the CTA revealed a "shaggy" lumen with 3.7 cm of mural thrombus and irregular ulcerations. In peripheral aneurysms, these findings are ominous; symptomatic cases present with distal embolization in 26% of patients and acute thrombosis in 15% [9]. Therefore, the imaging appearance alone justified a recommendation for aggressive management.

Management strategies

The primary goal of treatment is to prevent rupture and limb-threatening ischemia. Treatment of DFAAs includes endovascular exclusion, open reconstruction (bypass), or simple ligation.

Open Repair

This typically involves aneurysmectomy with either graft interposition or bypass [17]. However, in select cases where the superficial femoral artery is patent, simple ligation is a well-accepted alternative [7]. This is anatomically feasible due to the "cruciate anastomosis," a rich collateral network connecting the internal iliac and popliteal arteries, which maintains distal limb viability. Ligation avoids the longer operative times of reconstruction and the specific risks of graft infection, though all open approaches carry risks of lymphatic leak and wound complications [18].

Endovascular Repair

The use of covered stent-grafts (e.g., Viabahn) has been reported with good short-term results [6]. However, this approach is debated. The DFA was located in proximity to the hip joint, a zone of significant flexion. Consequently, the mechanical integrity of stents may be compromised, leading to high risks of fracture, kinking, and thrombosis which ultimately limits blood flow to the distal limb [18]. In particular, endovascular repair of the profunda femoris artery is associated with high rates of complications.

The Mayo Clinic does not recommend one method over the other, but all their cases were treated with aneurysmectomies [10]. Surgeons in Japan’s Okayama University also opted for the open surgical option, performing a combined aneurysmectomy and revascularization of an aneurysm 5x10 cm in size [19]. We detailed a similar treatment plan to our patient, opting for an aneurysmectomy with ligation of the side branches, but without revascularization due to the extensive collateralization. Surgery also carries risk. A recent case at Kansai Medical University had a patient undergo a simple ligation of a DFAA that started at 6.1 cm in size. Three years later, the aneurysm grew to 7.1 cm in diameter. An aneurysmorrhaphy was performed, a far more invasive procedure than hoped for, highlighting the importance of ligating all the branches during the first procedure [20]. Regardless, our patient refused treatment, notwithstanding our attempts at integrating him to a follow-up plan.

The management of this case highlights a significant ethical dilemma. Despite the high risk of a fatal rupture, the patient’s informed refusal of surgery was respected, as he demonstrated full cognitive capacity and mental competence. This underscores the importance of patient autonomy in modern clinical practice, even when faced with life-threatening conditions where surgical intervention is highly preventable.

## Conclusions

DFAA always presents a particular challenge, both diagnostically and therapeutically. Its rare appearance, misleading or even nonexistent symptoms, and the usually open and extensive surgical treatment demand extraordinary clinical expertise and excellent surgical skills. Our patient opted for a wait-and-watch approach. This underscores the fact that a patient can sometimes be reluctant to undergo high-risk therapy for a mainly asymptomatic condition. Our DFAA case is unique not only because of its diameter size and significant craniocaudal extension, but also because it is not associated with other aneurysms in other arterial segments and, most notably, was diagnosed in the context of multiple myeloma. We hope that this article provides some guidance for future diagnostic and handling strategies for this disease with regard to comorbidity and patient management.
